# HIF-1α Contributes to Proliferation and Invasiveness of Neuroblastoma Cells via SHH Signaling

**DOI:** 10.1371/journal.pone.0121115

**Published:** 2015-03-26

**Authors:** Sheng Chen, Min Zhang, Lili Xing, Yue Wang, Yongtao Xiao, Yeming Wu

**Affiliations:** 1 Department of Pediatric Surgery, Xinhua Hospital, School of Medicine, Shanghai Jiaotong University, Shanghai, China; 2 Shanghai Institute for Pediatric Research, Shanghai, China; University of Kansas Medical Center, UNITED STATES

## Abstract

The aim of this study was to investigate the effects of hypoxia-inducible factor-1α (HIF-1α) on the proliferation, migration and invasion of neuroblastoma (NB) cells and the mechanisms involved. We here initially used the real-time polymerase chain reaction (real-time PCR), Western blotting and immunohistochemistry (IHC) to detect the expression of HIF-1α and components of the sonic hedgehog (SHH) signaling pathway in NB cells and human specimens. Subsequently, cell proliferation, migration and invasion were analyzed using the cell counting assay, wound healing assay and Transwell system in two types of human NB cell lines, SH-SY5Y and IMR32. In addition, the role of HIF-1α in NB cells growth was determined in a xenograft nude mouse model. We found that the level of HIF-1α was significantly upregulated during NB progression and was associated with the expression of two components of SHH signaling, SHH and GLI1. We next indicated that the proliferation, migration and invasiveness of SH-SY5Y and IMR32 cells were significantly inhibited by HIF-1α knockdown, which was mediated by small interfering RNAs (siRNAs) targeting against its mRNA. Furthermore, the growth of NB cells in vivo was also suppressed by HIF-1α inhibition. Finally, the pro-migration and proliferative effects of HIF-1α could be reversed by disrupting SHH signaling. In conclusion, our results demonstrated that upregulation of HIF-1α in NB promotes proliferation, migration and invasiveness via SHH signaling.

## Introduction

Neuroblastoma (NB), which arises from neural crest precursors of the sympathetic nervous system, is one of the most common pediatric malignant solid tumors and accounts for 15% of childhood cancer deaths [1]. In contrast to great improvements in the survival rates for many other childhood cancers [2,3,4], the prognosis of advanced-stage NB remains poor despite multiple and intensive treatment regimens, such as surgery, chemotherapy, autologous stem cell rescue and radiotherapy.

Hypoxia is a common event in aggressive tumors that occurs when a tumor grows fast, and the blood supply is insufficient [5,6]. It is associated with local invasion, distant metastasis, and resistance to chemo- or radiotherapy in many malignant tumors [7,8]. An increased expression of hypoxia-inducible factor-1α (HIF-1α) is correlated with poor prognosis in some cancers, such as lung cancer [9,10], gastric cancer [11,12] and breast cancer [13,14].

In mammals, the hedgehog (HH) pathway is triggered by three related ligands, sonic hedgehog (SHH), Indian hedgehog (IHH) and desert hedgehog (DHH). The secreted ligands induce signaling by binding to Patched1 (PTCH1), inactivating PTCH1 and relieving inhibition of Smoothened (SMO), thus leading to the activation of glioma-associated oncogene (GLI) transcription factors [15].

HH signaling was reported to be deregulated in many cancers, including hepatocellular carcinoma, pancreatic cancer, gallbladder cancer, and lung cancer [16–19]. The SHH pathway was found to be persistently activated in NB cell lines and most primary NB specimens. Inhibition of the SHH pathway could induce apoptosis, block proliferation and decrease self-renewal ability in NB cells [20,21]. A recent study suggested that blockade of SHH signaling at the level of GLI transcription factors was an effective way to target high-risk NB [22]. These findings suggested that the SHH pathway might play a key role in the pathogenesis and progression of NB.

However, few studies have been reported concerning the correlation between HIF-1α and the SHH pathway in human cancers. During hypoxia, HIF-1α accumulation could introduce SHH rather than GLI activity in human pancreatic cancer cell lines. SHH secreted by pancreatic cancer cells could activate the hedgehog pathway and introduce a desmoplastic reaction in fibroblasts [23]. It is unclear whether HIF-1α could mediate biologic features such as proliferation, migration and invasion abilities in NB via the SHH signaling pathway. In the present study, we show that HIF-1α is responsible for the activation of the SHH pathway in NB, and it might regulate the abilities of proliferation, migration, invasiveness and tumorigenesis in NB via the SHH pathway.

## Materials and Methods

### Cell culture and reagents

The human NB cell lines SH-SY5Y and IMR32 were purchased from the Type Culture Collection of the Chinese Academy of Sciences, Shanghai, China. The cell lines were grown in high glucose Dulbecco's modified Eagle's medium (DMEM; Gibco, Grand Island, NY) containing 10% fetal bovine serum (FBS; Gibco, Grand Island, NY) at 37°C in a humidified 5% CO_2_ atmosphere. For hypoxic culture conditions, cells were incubated at 37°C in a humidified hypoxic chamber gassed with 1% O_2_, 5% CO_2_ and 94% N_2_.

### Cell viability assay

Cell counting kit-8 (CCK-8; Dojindo, Kumamoto, Japan) was used to determine NB cell proliferation based on the manufacturer’s protocol. In brief, cells were seeded in 96-well plates at a density of 5 × 10^3^ cells/well and were incubated under normoxia (20% O_2_) or hypoxia (1% O_2_). At the time of 0 h, 24 h, 48 h and 72 h, 10 μL of CCK-8 solution was added into each well and incubated for 1 hour at 37°C. The staining intensity in the medium was detected by measuring the absorbance (optical density, OD) at 450 nm.

### Wound healing assay

Cells were seeded in 12-well plates and grown to 80% confluency. The cell monolayers were scraped off with a sterile plastic tip and were cultured in DMEM with 1% FBS under normoxia (20% O_2_) or hypoxia (1% O_2_). Pictures were taken under microscope at 0 h and 24 h, and the distance of cell migration was assessed using NIS-Elements software (Nikon, Tokyo, Japan).

### Transwell invasion assay

The invasiveness of NB cells was assessed by their ability to pass through Matrigel (BD, Franklin Lakes, NJ)-coated Transwell inserts (Costar, Cambridge, MA) as described previously [24]. Briefly, the upper surface of the polycarbonic membranes (8.0-μm pore size) of the Transwell chambers was coated with Matrigel (1:6 diluted with DMEM). Cells (1×10^5^) in 100 μL of serum-free DMEM were seeded into the upper compartments of the chambers. Meanwhile, the lower compartments of the chambers were filled with 500 μL of DMEM containing 10% FBS. After 48 h, invasive cells that had migrated from Matrigel to the lower surface of the filters were fixed in 0.1% paraformaldehyde, stained with crystal violet (Beyotime, Haimen, China), and counted under an inverted microscope at ×200 magnification. Cell invasion was expressed as the average number of cells counted in 3 randomly selected visual fields per filter.

### Small interference RNAs (siRNAs) and transfection

Three siRNAs targeting GLI1 (named siRNA1, siRNA2 and siRNA3, respectively) and negative control (NC) siRNA were purchased from GenePharma Co., Ltd. (Shanghai, China). The sequences of GLI1 siRNAs and NC siRNA are shown in S1 Table. The siRNAs were diluted to produce a final concentration of 40 nM in Opti-Mem (Invitrogen, Carlsbad, CA). Transient transfections were performed using Lipofectamine 2000 (Invitrogen, Carlsbad, CA) according to the manufacturer’s instructions.

### Real-time PCR analysis

Total RNA was extracted with TRIzol reagent (Invitrogen, Carlsbad, CA) and reverse-transcribed to cDNA using the PrimeScript RT reagent Kit (Takara Bio, Dalian, China) according to the manufacturer’s protocol. The primer sequences were designed online (http://www.ncbi.nlm.nih.gov/tools/primer-blast) and synthesized by Sangon Biotech (Shanghai, China). All of the primer sequences are provided in S2 Table. Quantitative PCR was performed using a 7500 Real-time PCR System (Applied Biosystems, Foster City, CA) and the Power SYBR Master Mix (Applied Biosystems, Foster City, CA) following the manufacturer's instructions. The relative mRNA expression level was calculated using the 2^-ΔΔCt^ method with the Ct values normalized using β-actin as an internal control.

### Western blotting

After the indicated treatment, cells were lysed with RIPA buffer (Thermo, Rockford, IL) mixed with protease inhibitor cocktail (Thermo, Rockford, IL). Protein concentrations were determined using Pierce BCA Protein Assay Kit (Thermo, Rockford, IL). Equal amounts of protein samples (30 μg/well) were separated electrophoretically by SDS-PAGE, and transferred to PVDF membranes (Roche, Mannheim, Germany). The membranes were blocked for 1 hour in PBS-Tween 20 with 5% bovine serum albumin. Thereafter, the blots were probed with primary antibodies against HIF-1α (1:1000), GLI1 (1:500), β-actin (1:1000; all from Abcam, Cambridge, MA), SHH (1:1000; Cell Signaling Technology, Danvers, MA), and PTCH1 (1:500; Santa Cruz Biotechnology, Santa Cruz, CA). The blots were washed in PBS-Tween 20, incubated with the appropriate secondary antibodies (horseradish peroxidase conjugated), and visualized using Super Signal West Pico (Thermo, Rockford, IL).

### Lentiviral vectors

The HIF-1α expression lentiviral vector Lenti-GFP- HIF-1α, control lentivirus Lenti-GFP, lentiviral vector Lenti-GFP-HIF-1α siRNA and control lentivirus Lenti-GFP-scrambled siRNA were constructed by GeneChem Biotechnology (Shanghai, China). Briefly, the full sequence of human HIF-1α (NM_001530) was cloned and connected to the lentiviral vector pGCL-GFP with the pUbi promoter. Three siRNAs targeting the human HIF-1α gene were designed. The siRNA expression plasmids (named siRNA1, siRNA2 and siRNA3, respectively) were constructed by inserting siRNA sequences into the pGCL-GFP vector with the phU6 promoter. A lentiviral vector containing NC siRNA was constructed by a similar process. The sequences of HIF-1α siRNAs and NC siRNA are provided in S3 Table. The constructs were verified by restriction enzyme analysis and DNA sequencing. The production, concentration and titration of lentivirus were carried out as described previously [25]. The titer of the lentiviral stock was 2×10^9^ titer units (TU)/mL. Cells in a 6-well plate at a confluency of 50% were transfected by virus particles at a multiplicity of infection (MOI) of 50 for 24 h. The transfection rate was determined by fluorescence microscopy (Nikon, Tokyo, Japan) and Western blotting. Cells were used for the following experiments 72 hours after transfection.

### Subcutaneous tumor model

Four week-old male athymic Balb/c nude mice were purchased from Shanghai Slac Laboratory Animal Co., Ltd. and housed in individually ventilated microisolator cages. Nude mice were divided into 5 groups of 5 mice each. Four groups were injected subcutaneously in the flank with 5 ×10^6^ SH-SY5Y cells transduced with Lenti-HIF-1α, Lenti-GFP, Lenti-HIF-1α siRNA or Lent**i**-scrambled siRNA in 0.1 mL of DMEM. The other group of mice were injected with the same amount of cells transduced with Lenti-HIF-1α, and were given GANT61 50 mg/kg daily through gastric feeding on the 1st day after injection until they were sacrificed. The reference for the dose of GANT61 can be found in Wickström's work [22]. Tumor sizes were determined with calipers every 4 days by measuring the length and width. Tumor volumes were calculated according to the following formula: volume (mm^3^) = (length · width^2^) / 2 [26]. Twenty-eight days after tumor cell injection, mice were sacrificed and tumor xenografts were removed, weighed, and fixed in formalin and stored at 4°C. Animal experiments were approved by the Animal Care and Use Committee of Xinhua Hospital.

### Immunohistochemistry (IHC)

The use of paraffin-embedded human NB specimens was approved by the ethics committee of Xinhua Hospital (approval number XHEC-D-2036). The study cohort consisted of 71 NB samples obtained between January 2008 and December 2013. All the parents of patients provided written informed consent to participate in this study. The consent procedure was also approved by the ethics committee of Xinhua Hospital. The clinical information of the patients is presented in S4 Table. Formalin-fixed xenograft samples were embedded in paraffin and cut into 4 μm thick sections. The immunohistochemical study was performed using a standard two-step peroxidase technique as described previously [27]. Slides were dewaxed, rehydrated and processed for antigen retrieval. Endogenous peroxidase was quenched with 0.03% hydrogen peroxide for 20 min, and nonspecific reaction was blocked with 5% goat serum for 30 min. Subsequently sections were incubated with primary antibody against HIF-1α (1:200; Abcam, Cambridge, MA), SHH (1:200; Santa Cruz Biotechnology, Santa Cruz, CA), PTCH1 (1:100; Santa Cruz Biotechnology, Santa Cruz, CA) or GLI1 (1:100; Abcam, Cambridge, MA) at 4°C overnight. After washing, the slides were incubated with horseradish peroxidase-labeled secondary antibody at 37°C for 1 h. Finally, they were incubated in phosphate buffered saline containing diaminobenzidine (DAB) for 5 min and then examined on a microscope.

Immunohistochemical staining was scored semiquantitatively by 3 independent pathologists unaware of the experiment and clinical data. The scoring criterion was according to the percentage of positively stained cells as follows: 0 (0–5%); 1 (6–25%); 2 (26–50%); 3(51–75%); 4(76–100%). If at least 2 of the pathologists agreed with the scores, the result was accepted. The scoring system was applied to both xenograft and clinical NB samples.

### Statistical analysis

Statistical analysis was performed using SPSS 16.0 statistical software. Data are presented as means ± standard deviation (SD) of three independent experiments. The association among the expression levels of HIF-lα and SHH signals in human NB tissues and tumor stages was analyzed using Spearman rank correlation. Statistical comparison of HIF-1α and SHH signals expression in human specimens between clinicopathological factors such as tumor differentiation and lymph node involvement was assessed by chi-squared test. Other comparisons between the two groups were performed using two-sided Student's t test. For all tests, a *P* value less than 0.05 was considered statistically significant.

## Results

### HIF-1α correlates with the hedgehog signaling pathway and tumor stage in clinical NB specimens

**Fig 1:**
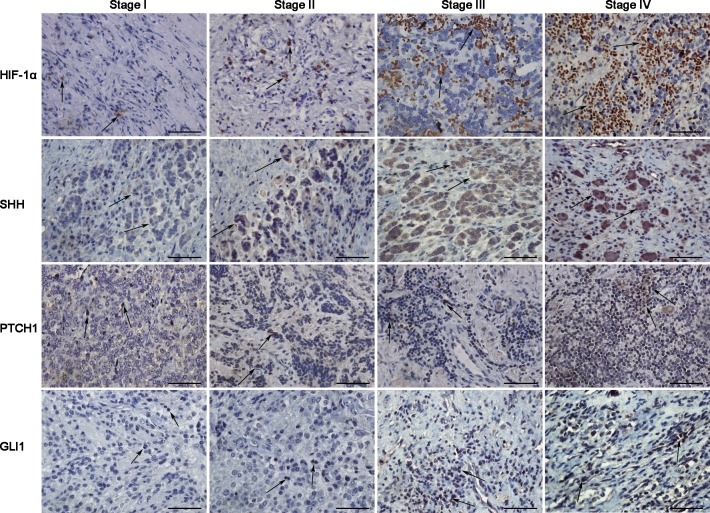
Representative immunohistochemical staining images of HIF-1α and SHH pathway components in human NB samples. Compared with early-stage (stage I or II) NB, advanced-stage (stage III or IV) tumors tended to show stronger staining for HIF-1α and SHH signals. Spearman rank correlation indicated that HIF-1α was positively correlated with SHH and GLI1 staining. HIF-1α, SHH and GLI1 expression levels were related with tumor stage. All ×400 magnification. Scale bars, 200 μm. Arrows, positive cells.

The results of IHC are presented in Table 1. Positive expression of HIF-1α was found in 57.7% of all the tumor samples from all clinical stages, and the degree of positive staining for SHH, PTCH1 and GLI1 was 64.8%, 46.5%, and 54.9%, respectively (Table 1; S4 Table). Spearman rank correlation showed a positive association between HIF-1α and the SHH expression levels (Spearman rho = 0.445; *P* < 0.0001). HIF-1α staining intensity was also positively correlated with GLI1 (Spearman rho = 0.431; *P* < 0.0001). However, we did not find a correlation between HIF-1α and PTCH1 (Spearman rho = 0.167; *P* = 0.163). In addition, our data showed HIF-1α, SHH and GLI1 expression levels were related to International Neuroblastoma Staging System (INSS) stages (Fig. 1) (Spearman rho = 0.415, *P* < 0.0001; Spearman rho = 0.487, *P* < 0.0001; Spearman rho = 0.347, *P* = 0.003). That is, advanced stage tumors frequently expressed high levels of these three proteins. Furthermore, lymph node metastasis was found in 34 patients (48%). Tumors with lymph node metastasis showed higher frequency of SHH and GLI1 positivity (SHH: 79% vs. 51%, *P* = 0.013; GLI1: 68% vs. 43%, *P* = 0.039). Finally, HIF-1α and GLI1 staining rates were found to be markedly higher in poorly-differentiated tumors than in well-differentiated ones (HIF-1α: 73% vs. 39%, respectively, *P* = 0.004; GLI1: 68% vs. 39%, respectively, *P* = 0.016).

**Table 1:** Expression of SHH pathway proteins in NB samples at different stages and differentiation conditions.

	All cases	HIF-1α positivity (%)	SHH positivity (%)	PTCH1 positivity (%)	GLI1 positivity (%)
INSS stage					
I	18	5 (28)	7 (39)	6 (33)	4 (22)
II	17	7 (41)	9 (53)	10 (59)	13 (76) **
III	12	11 (92) ** ^,^ ^##^	10 (83) *	3 (25)	8 (67) *
IV	24	18 (75) ** ^,^ ^#^	20 (83) ** ^,^ ^#^	14 (58)	14 (58) *
Lymph node metastasis					
No	37	18 (49)	19 (51)	15 (41)	16 (43)
Yes	34	23 (68)	27 (79)^Δ^	18 (53)	23 (68) ^Δ^
Differentiation					
Well	31	12 (39)	18 (58)	12 (39)	12 ((39)
Poor	40	29 (73) ^§§^	28 (70)	21 (53)	27 (68) ^§^

* *p*<0.05 versus stage I;

** *p*<0.01 versus stage I;

^#^ *p*<0.05 versus stage II;

^##^ *p*<0.01 versus stage II;

^Δ^ *p*<0.05 versus the without lymph node metastasis group;

^§^ *p*<0.05 versus the well-differentiated group;

^§§^ *p*<0.01 versus the well-differentiated group.

### Effects of HIF-1α on proliferation of NB cells in vitro

**Fig 2:**
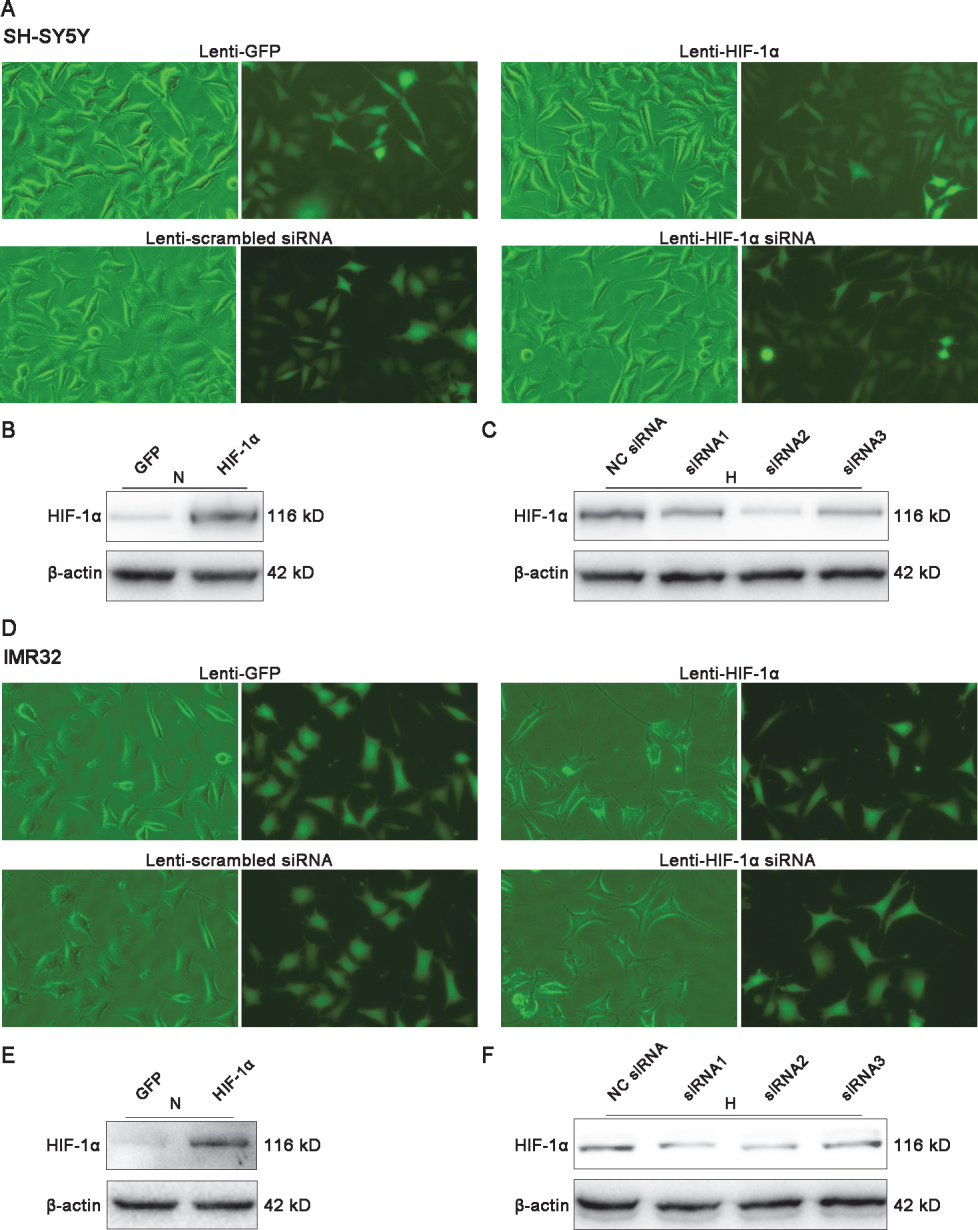
Overexpression and knockdown of HIF-lα in NB cells by lentiviral vectors. (A, D) SH-SY5Y and IMR32 cells were transfected with lentiviral vectors and observed by fluorescence microscopy after 72 h. More than 90% of the cells expressed green fluorescent protein (GFP). Magnification, ×200. (B, E) Western blot analysis showed significantly higher HIF-lα expression in SH-SY5Y and IMR32 cells transfected by Lenti-HIF-lα than in cells transfected by Lenti-GFP after 72 h under normoxia. β-actin was used as a loading control. (C, F) SH-SY5Y and IMR32 cells were transfected with Lenti-scrambled siRNA and three different siRNAs targeting HIF-lα. After a 72-h-transduction, an additional 8-h-hypoxic treatment was needed before Western blotting. siRNA2 was proven to be the most effective one of the three siRNAs. β-actin was used as a loading control. N, normoxia; H, hypoxia.

To determine the role of HIF-lα in NB cells, we transfected cells with lentiviral vectors that overexpress or knockdown HIF-lα. More than 90% of SH-SY5Y and IMR32 cells presented green fluorescence after the transfection, indicating that the vast majority of these cells had been successfully transfected (Fig. 2A and D). Western blotting showed a marked increase in HIF-lα under normoxic conditions 72 h after Lenti-HIF-lα transfection (Fig. 2B and E). For cells transfected with Lenti-HIF-lα siRNAs, an additional 8 h of hypoxic treatment was needed before Western blotting because HIF-lα expression was too low under normoxia. Lenti-HIF-lα siRNA2 was more effective than the other two (Fig. 2C and F), so we chose it for subsequent experiments.

**Fig 3:**
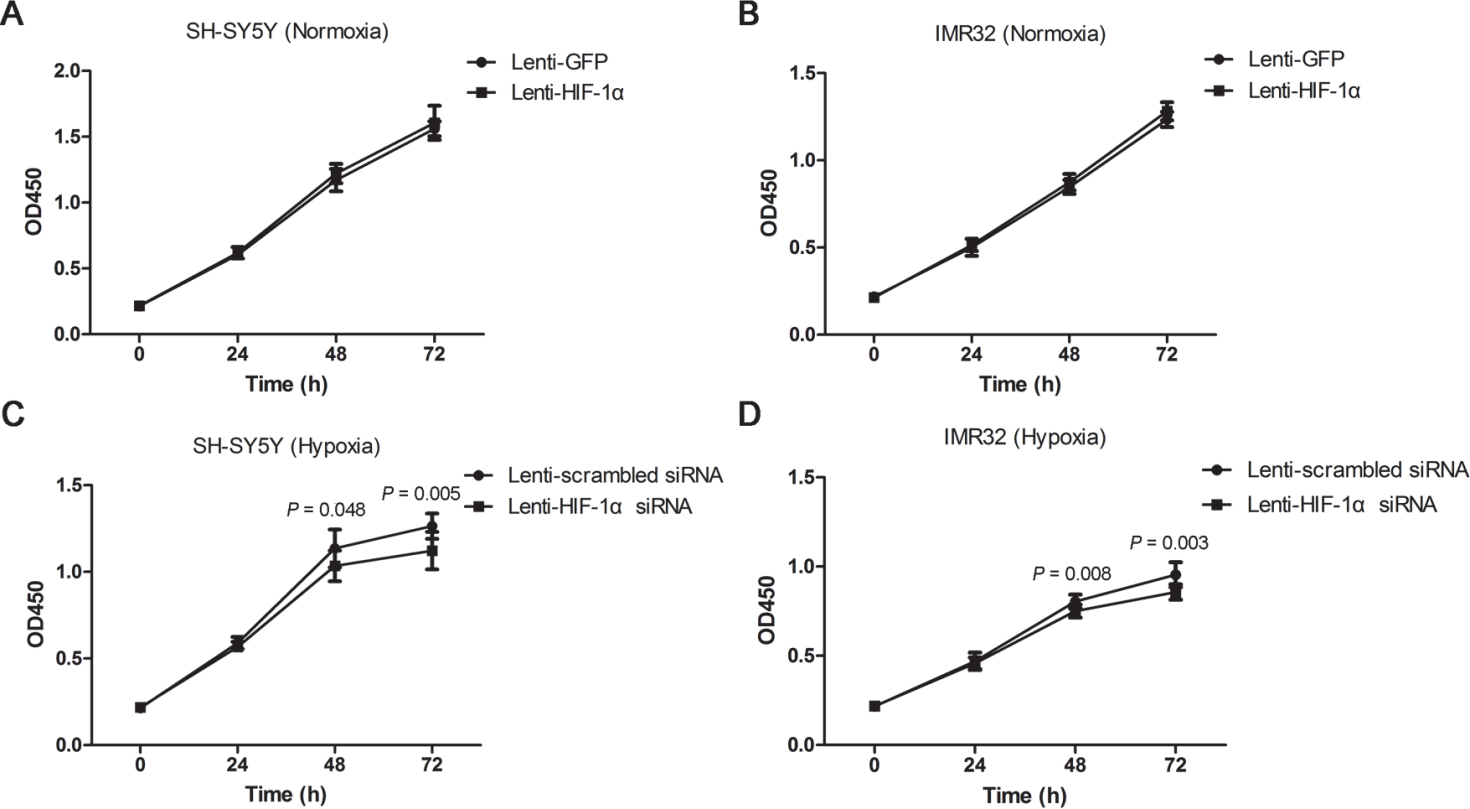
Effect of HIF-1α on the proliferation of NB cells. (A, B) The CCK-8 assay showed that overexpression of HIF-1α using a lentiviral vector did not promote cell proliferation in either SH-SY5Y or IMR32 cells in normoxia (n = 9). (C, D) Lentiviral vector-mediated HIF-lα knockdown inhibited cell proliferation in SH-SY5Y and IMR32 cells under hypoxia (n = 9). Data are expressed as means ± SD.

We found that overexpression of HIF-1α could not promote the growth of both SH-SY5Y and IMR32 cell lines under normoxia during the 72-h culture (*P* = 0.349 and *P* = 0.054, respectively) (Fig. 3A and B). However, compared with the control, NB cells transfected with Lenti-HIF-lα siRNA underwent marked growth inhibition under hypoxic conditions at 72 h (*P* = 0.005 and *P* = 0.003, respectively) (Fig. 3C and D).

### HIF-1α promotes the migration of NB cells in vitro

**Fig 4:**
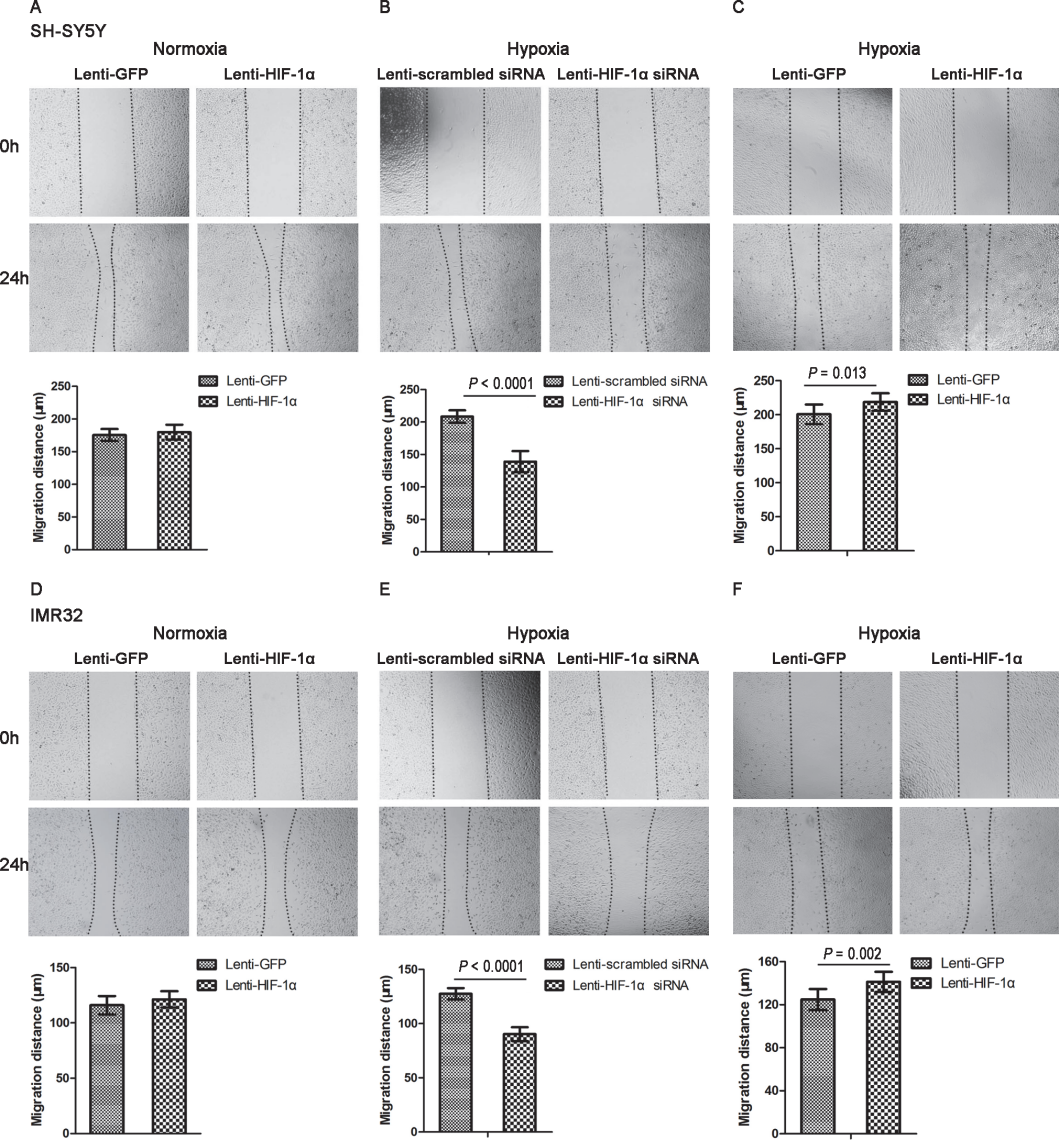
Effect of HIF-1α on the migration of NB cells. (A, D) The wound healing assay showed that HIF-1α overexpression did not increase migration in SH-SY5Y and IMR32 cells under normoxia (n = 9). (B, E) HIF-1α knockdown decreased migration in both cell lines under hypoxia (n = 9). (C, F) HIF-1α overexpression increased migration in both cell lines under hypoxia (n = 9). All ×40 magnification. Data are expressed as means ± SD.

Both SH-SY5Y and IMR32 cells transfected with Lenti-HIF-lα showed similar migration distance to cells transfected with Lenti-GFP after 24 h of normoxic culture, indicating that HIF-lα overexpression did not have an effect on the migration ability of NB cells under normoxia (*P* = 0.380 and *P* = 0.169, respectively) (Fig. 4A and D). However, their migration distance was significantly reduced by 33% and 29%, respectively, by knockdown of HIF-1α under hypoxic conditions (both *P* < 0.0001) (Fig. 4B and E). Interestingly, the migration distance increased by 9% and 13%, respectively, by overexpression of HIF-lα under hypoxia (*P* = 0.013 and *P* = 0.002, respectively) (Fig. 4C and F). The results of the wound healing assay indicate that HIF-1α promotes NB cell migration under hypoxia but not under normoxia.

### HIF-1α promotes the invasive abilities of NB cells in vitro

**Fig 5:**
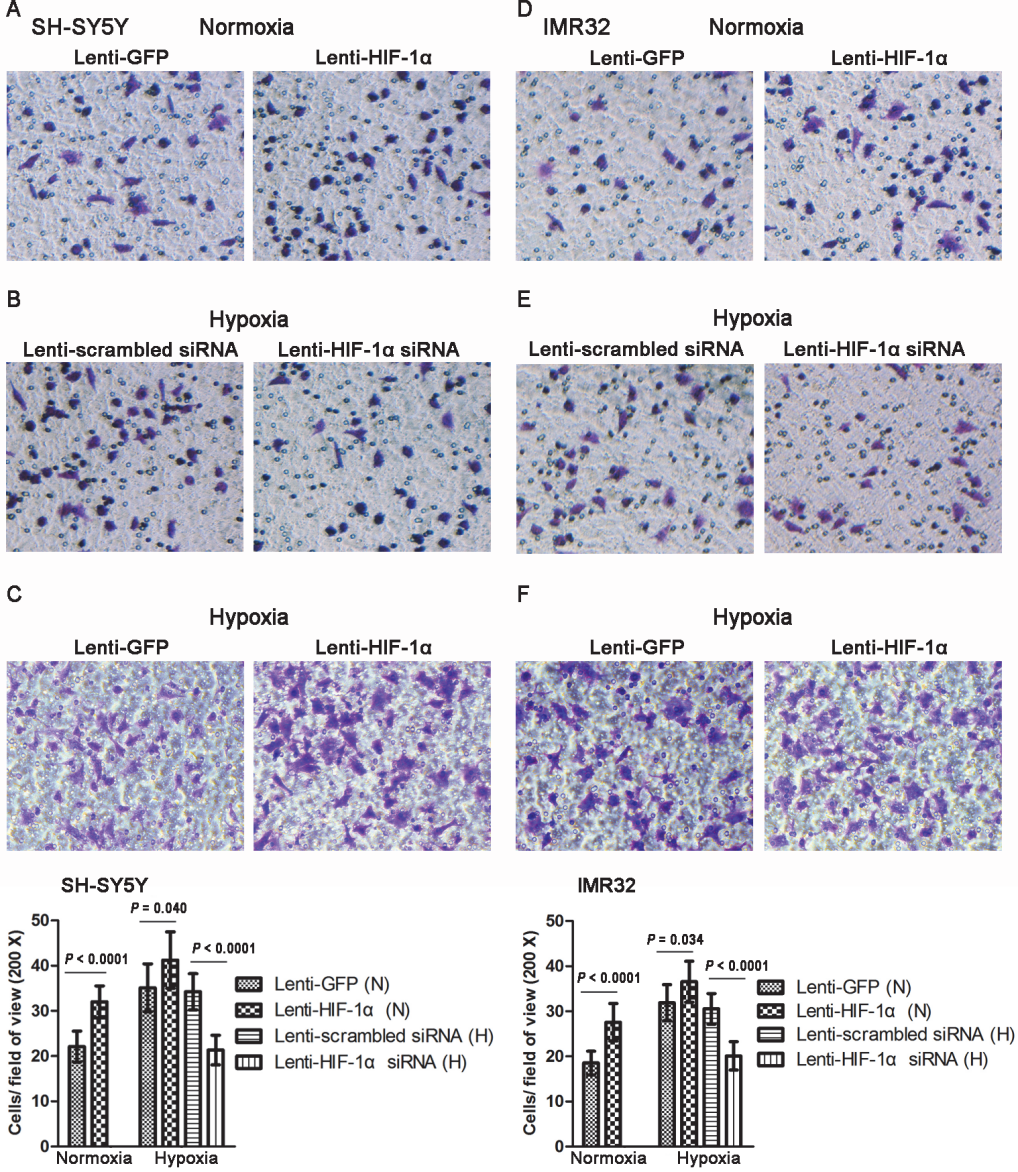
Effect of HIF-1α on the invasion of NB cells. (A, D) The Transwell assay showed that HIF-1α overexpression enhanced invasion in SH-SY5Y and IMR32 cells under normoxia (n = 9). (B, E) HIF-1α knockdown reduced invasion in both cell lines under hypoxia (n = 9). (C, F) HIF-1α overexpression enhanced invasion in both cell lines under hypoxia (n = 9). All ×200 magnification. Data are expressed as means ± SD.

To evaluate the effect of HIF-1α on the invasive abilities of NB cells under normoxic and hypoxic conditions, we used a Transwell assay. Compared with the Lenti-GFP control group, the number of cells that passed through the Matrigel-coated membranes increased approximately 45% in SH-SY5Y and 48% in IMR32 cells transfected with Lenti-HIF-1α under normoxic conditions (both *P* < 0.0001) (Fig. 5A and D). However, knockdown of HIF-1α caused 38% and 34% reduction in SH-SY5Y and IMR32 cell invasion respectively under hypoxia (both *P* < 0.0001) (Fig. 5B and E). Furthermore, the invasive abilities increased by 17% and 15%, respectively, by overexpression of HIF-lα under hypoxic conditions (*P* = 0.040 and *P* = 0.034, respectively) (Fig. 5C and F). The Transwell assay shows that HIF-1α could effectively enhance the invasive abilities in SH-SY5Y and IMR32 cells under both normoxia and hypoxia.

### HIF-1α regulates the SHH signaling pathway in NB cells

**Fig 6:**
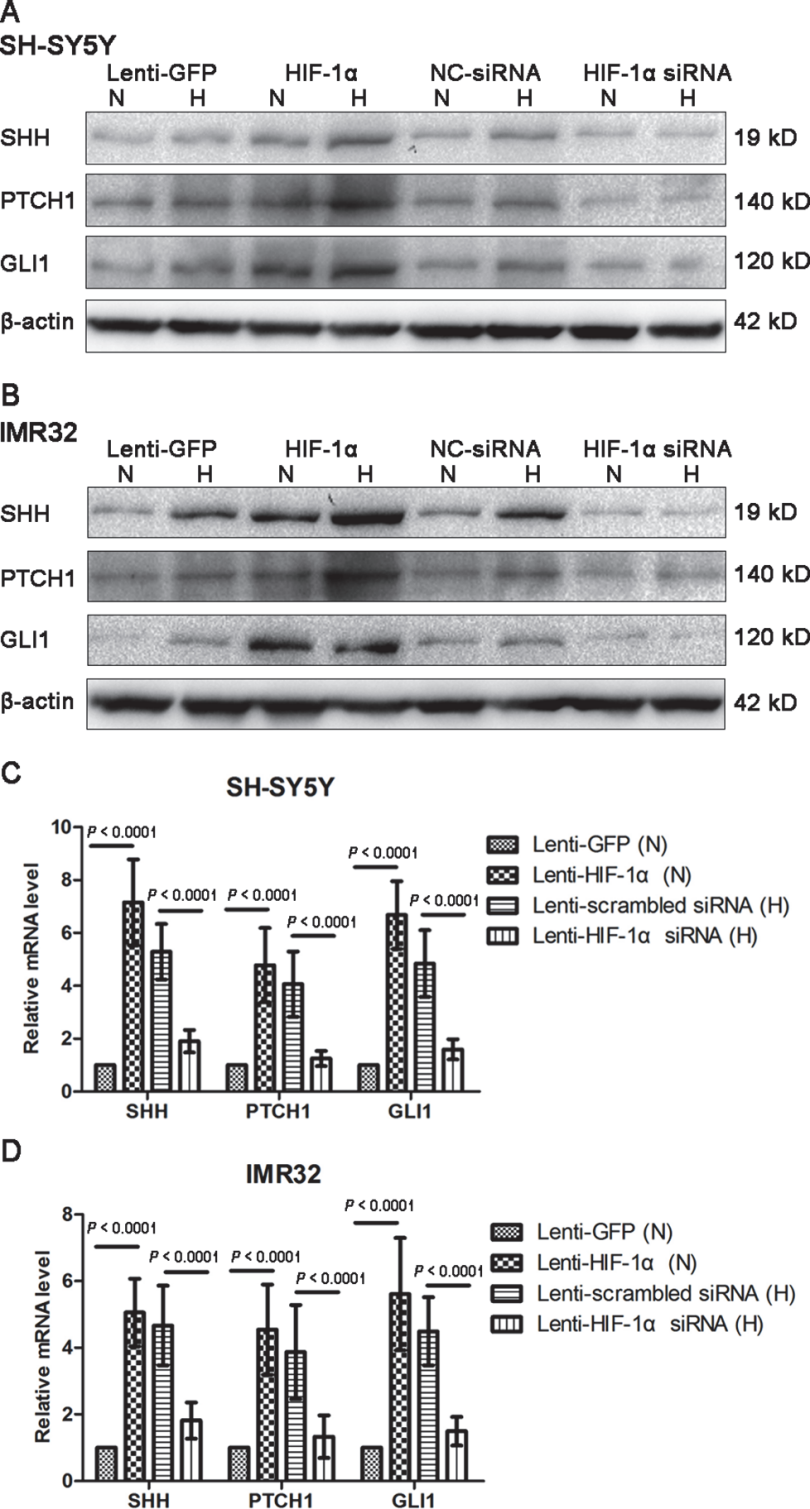
HIF-1α regulates the SHH signaling pathway in NB cells. (A, B) Western blotting results showed that the protein levels of SHH, PTCH1 and GLI1 were positively associated with the levels of HIF-1α in NB cells under normoxia and hypoxia. β-actin was used as a loading control. (C, D) The mRNA expression of SHH, PTCH1 and GLI1 were estimated by real-time PCR. The results indicated that the mRNA expression of SHH pathway components were also regulated by HIF-1α (n = 9). The expression of each target gene was quantified using β-actin as a normalization control. Data are expressed as means ± SD. N, normoxia; H, hypoxia.

To determine the regulation effect of HIF-1α on the SHH pathway, the important components of the signaling pathway such as SHH, PTCH1 and GLI1 were detected by Western blotting in SH-SY5Y and IMR32 cells overexpressing or being knocked down of HIF-1α. Cells transfected with Lenti-scrambled siRNA or Lenti-HIF-1α siRNA were incubated for 8 h under hypoxia before lysis because of the extremely low expression of HIF-1α under normoxia. We found that the expression levels of SHH, PTCH1 and GLI1 were strongly linked with the level of HIF-1α. Their levels were significantly upregulated in both cell lines overexpressing HIF-1α and were markedly downregulated in HIF-1α knockdown cells (Fig. 6A and B). Because the expression of HIF-1α is increased under hypoxia, there was a higher expression of the three important components of the SHH pathway in cells treated under hypoxia than those treated under normoxia (Fig. 6A and B). The results of Western blotting suggested that HIF-1α is an upstream regulator of the SHH pathway.

To identify whether the increase in SHH signals resulted from de novo protein synthesis instead of increased protein processing, we detected their mRNA levels in SH-SY5Y and IMR32 cells. Cells transfected with Lenti-scrambled siRNA or Lenti-HIF-1α siRNA were also incubated for 8 h under hypoxia before RNA extraction. Real-time PCR analysis showed an increase in SHH, PTCH1 and GLI1 transcripts levels in both cell lines transfected with Lenti-HIF-1α under normoxia compared with the control (all *P* < 0.0001) (Fig. 6C and D). As expected, HIF-1α knockdown reduced SHH, PTCH1 and GLI1 transcripts levels in SH-SY5Y and IMR32 cells under hypoxia (all *P* < 0.0001) (Fig. 6C and D).The results of real-time PCR were consistent with those of Western blotting, suggesting that HIF-1α also regulated the SHH pathway through transcript levels in NB cells, further influencing protein expression.

### GLI1 knockdown reverses the positive effect of HIF-1α on the biological behavior of NB cells

**Fig 7:**
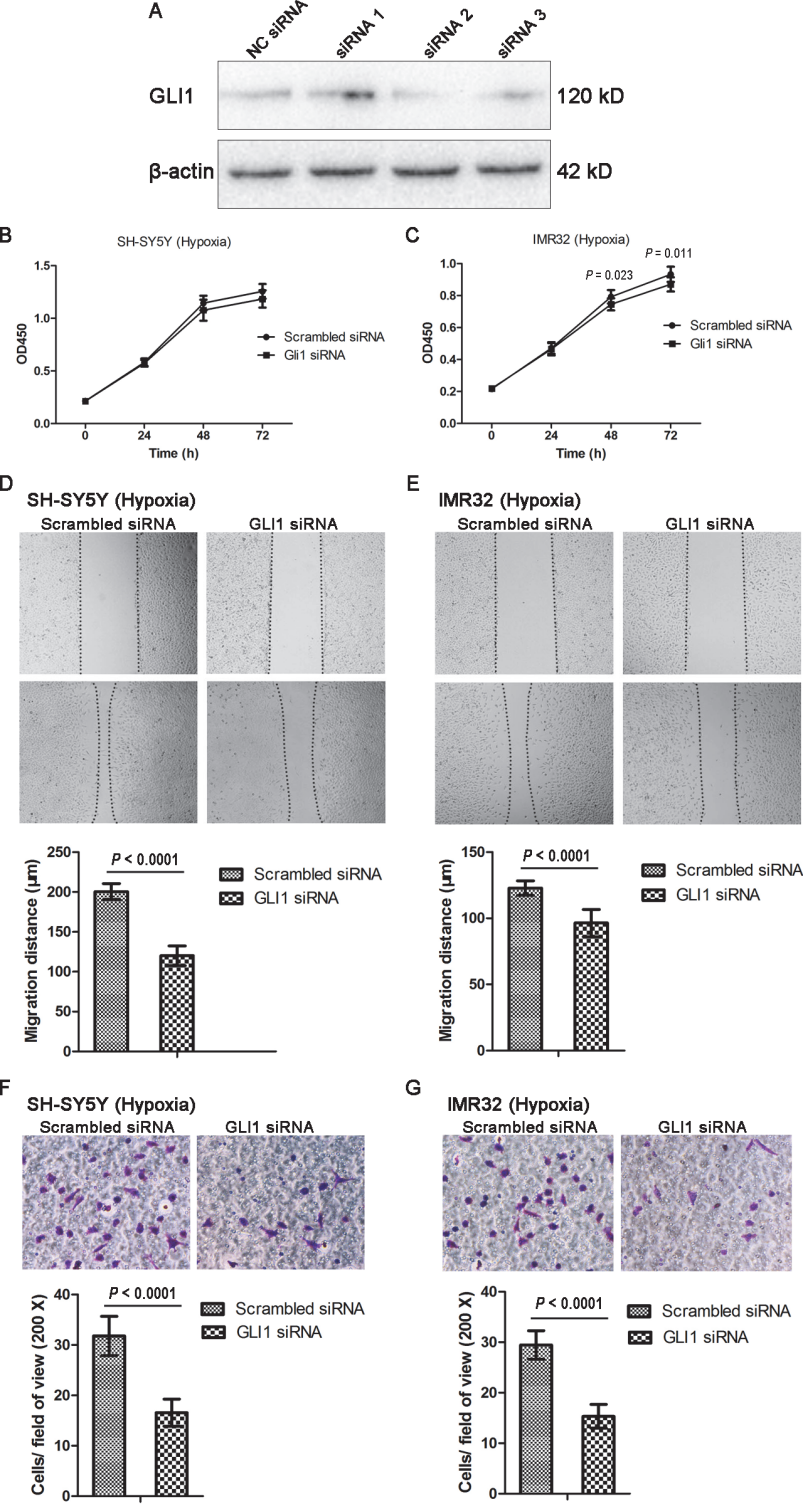
GLI1 knockdown inhibits the cell proliferation, migration and invasion abilities in NB cells under hypoxic conditions. (A) Three siRNAs targeting GLI1 and NC siRNA were transfected into SH-SY5Y cells. Western blotting indicated that siRNA2 provided the highest inhibition efficiency. (B, C) GLI1 knockdown significantly inhibited proliferation in IMR32 cells, but only led to a slight reduction in proliferation in SH-SY5Y cells (n = 9). (D, E) Decreased cell migration was observed in both cell lines transfected with GLI1 siRNA (n = 9). Magnification, ×40. (F, G) Invasion ability was reduced in both cell lines transfected with GLI1 siRNA (n = 9). Magnification, ×200. Data are expressed as means ± SD.

SH-SY5Y cells were transfected with three siRNAs targeting GLI1 and NC siRNA according to the method mentioned above. Western blotting indicated that GLI1 siRNA2 achieved the highest knockdown efficacy among the three pairs of siRNAs, so we selected it for subsequent studies (Fig. 7A). To determine whether the inhibition of SHH signaling mediates the cell proliferation, migration and invasion abilities of NB cells expressing high levels of HIF-1α, we transfected SH-SY5Y and IMR32 cells with GLI1 siRNA or scrambled siRNA as control and repeated the above-mentioned experiments under hypoxic conditions. In IMR32 cells, the CCK-8 assay showed that cell proliferation was markedly inhibited in GLI1 knockdown cells at 48 h and 72 h compared with control cells (*P* = 0.023 for 48 h and *P* = 0.011 for 72 h) (Fig. 7C). However, there was a slight proliferation inhibition in SH-SY5Y cells transfected with GLI1 siRNA, but with no significant difference (*P* = 0.114 for 48 h and *P* = 0.059 for 72 h) (Fig. 7B). The wound healing and Transwell assays demonstrated that, compared with control cells, both the migration and invasion abilities were reduced in both cell lines transfected with GLI1 siRNA (all *P* < 0.0001) (Fig. 7D-G). These results support a mechanism by which SHH signaling inhibition mediates the effects of HIF-lα on NB cells.

**Fig 8:**
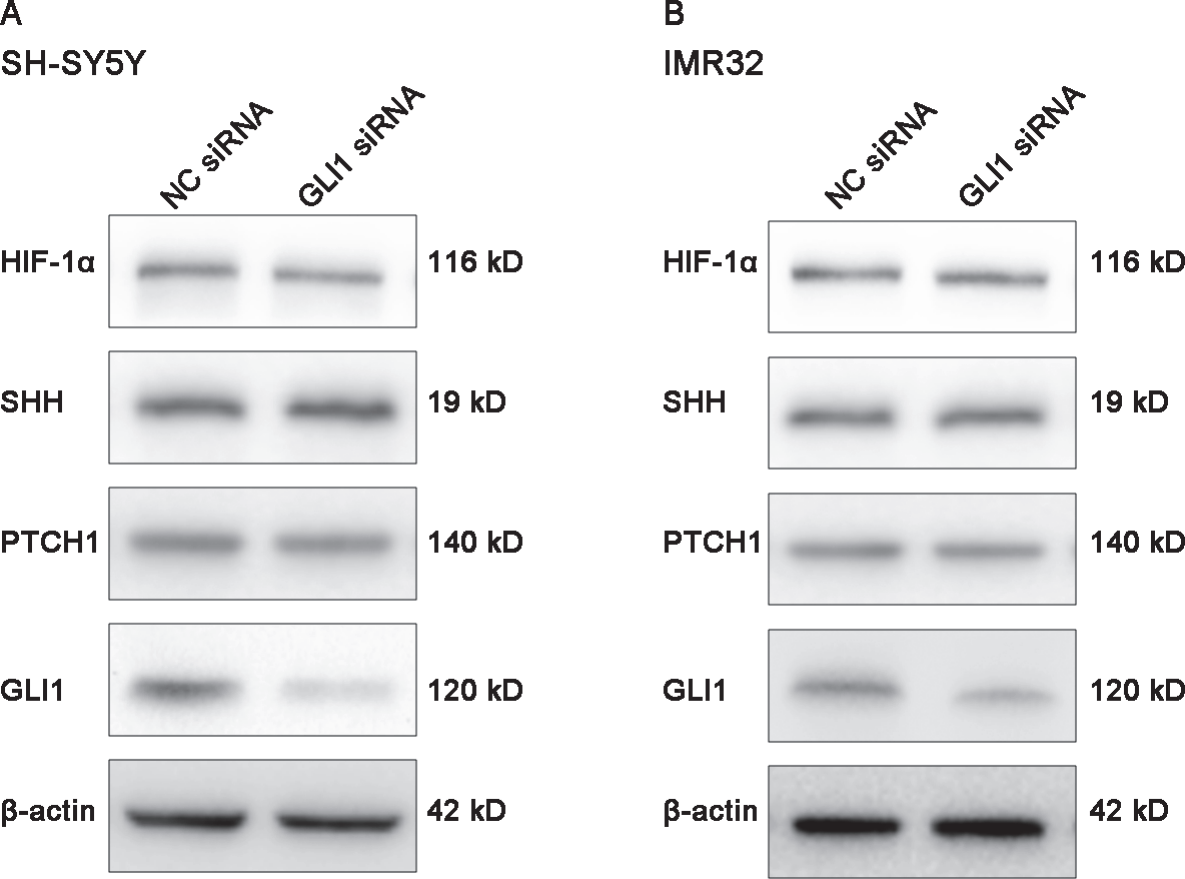
Effect of GLI1 knockdown on the expression levels of HIF-1α and SHH pathway components in NB cells. (A, B) Western blotting results showed that the protein levels of HIF-1α, SHH and PTCH1 did not change in SH-SY5Y and IMR32 cells when GLI1 was knocked down under hypoxia. β-actin was used as a loading control.

In addition, we detected the expression levels of HIF-1α and SHH pathway components in NB cells transfected with GLI1 siRNA and NC siRNA by Western blotting. Cells were incubated for 8 h under hypoxia before lysis. The results showed that the levels of HIF-1α, SHH and PTCH1 did not change in both cell lines when GLI1 was knocked down (Fig. 8A and B), which further demonstrated that GLI1 was a downstream effector of the HIF-1α-regulated SHH pathway.

### HIF-1α enhances tumor growth in xenografted NB tumors

**Fig 9:**
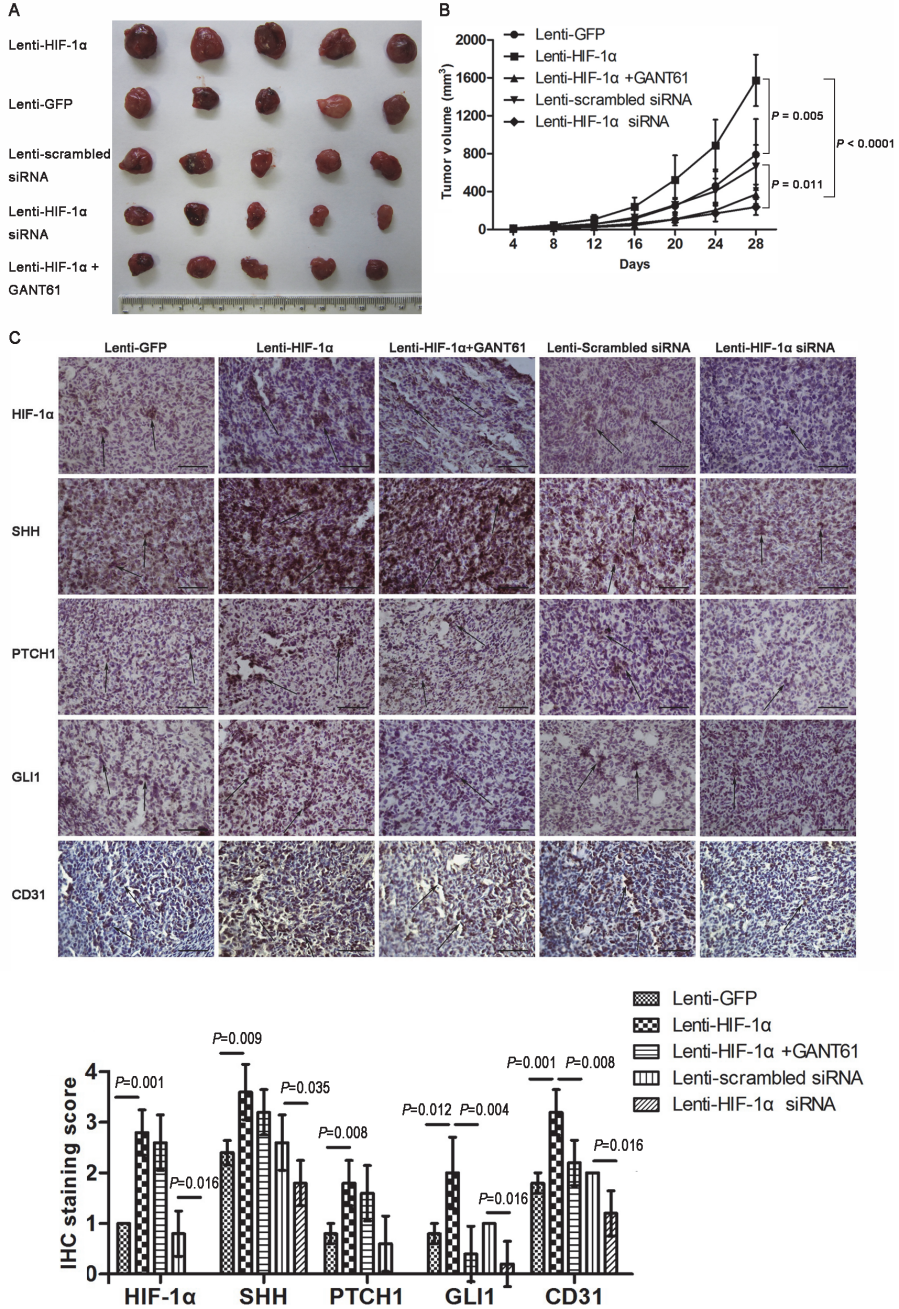
HIF-1α regulates NB growth and the SHH pathway in vivo. (A) Representative xenografts dissected from different groups of nude mice are shown. (B) The volume of subcutaneous xenografts formed by SH-SY5Y cells stably infected with the indicated lentiviruses is illustrated by growth curves (n = 5). Compared with the control, cells overexpressing HIF-lα formed significantly larger tumors. By contrast, the tumor weight in the HIF-lα knockdown group was significantly reduced. GANT61 could reverse the pro-growth effect of HIF-lα. (C) Immunohistochemical staining of HIF-1α, SHH pathway components and CD31 in xenografts. The statistical results showed that the expression levels of SHH pathway components and angiogenesis in xenografts were correlated with HIF-lα (n = 5). In the bar graph, 4 bars were indicated for HIF-1α and PTCH1 as mean scores for both subgroups were 0. Magnification, ×400. Scale bars, 200 μm. Arrows, positive cells.

To further demonstrate the regulatory effect of HIF-1α we found in vitro, we established a tumor xenograft model. SH-SY5Y cells transfected with Lenti-HIF-lα formed significantly larger tumors in mice compared with cells transfected with control virus (*P* = 0.005) (Fig. 9A and B). By contrast, the tumor volume of NB xenografts transduced with Lenti-HIF-lα siRNA was smaller than that of the control (*P* = 0.011) (Fig. 9A and B). GANT61, a small molecule antagonist of GLI, was delivered in mice carrying xenografts overexpressing HIF-1α to determine whether inhibition of the SHH pathway could reverse the pro-growth role of HIF-1α in xenografts. In mice injected with HIF-1α-overexpressing cells, but given GANT61, the tumor volume was remarkably smaller (*P* < 0.0001) (Fig. 9A and B), suggesting that the positive effect of HIF-1α on tumor growth was antagonized via inhibiting the SHH pathway.

### HIF-1α regulates SHH signaling in vivo

Xenografts from mice were detected for the expression levels of HIF-lα and SHH pathway components by IHC. As expected, immunohistochemical staining showed that HIF-lα was overexpressed in tumor tissue derived from mice in the Lenti-HIF1α group (*P* = 0.001) and downregulated in the Lenti-HIF1α-siRNA group (*P* = 0.016) (Fig. 9C). Compared with the control, SHH, PTCH1, GLI1 and CD31 were upregulated in the HIF-lα-overexpression group (*P* = 0.009 for SHH; *P* = 0.008 for PTCH1; *P* = 0.012 for GLI1; *P* = 0.001 for CD31) and downregulated in the HIF-lα knockdown group (*P* = 0.035 for SHH; *P* = 0.07 for PTCH1; *P* = 0.016 for GLI1; *P* = 0.016 for CD31) (Fig. 9C). However, compared with the HIF-lα overexpression group, the HIF-lα overexpression plus GANT61 treatment group showed similar levels of SHH and PTCH1 (*P* = 0.242 for SHH; *P* = 0.545 for PTCH1), but lower levels of GLI1 and CD31 (*P* = 0.004 for GLI1; *P* = 0.008 for CD31) (Fig. 9C). These results further demonstrated that HIF-lα regulated the growth and angiogenesis of NB through the SHH pathway.

## Discussion

Hypoxia has been reported previously to induce a rapid increase in the expression of SHH and PTCH1 in various organs of adult mice as well as in rat cardiomyoblast cells [28]. Whether HIF-1α regulates the biological behaviors of cancers via SHH signaling is a promising research direction. Nevertheless, there are few studies referring to this topic. We here show that HIF-1α regulates the SHH pathway in NB and further influences the growth and progression of the tumor. Initially, our data showed that high expression levels of HIF-1α in clinical tumor specimens were correlated with advanced tumor stage and poor differentiation, suggesting that HIF-1α is a potential biomarker for NB progression (Table 1 and Fig. 1). Furthermore, HIF-1α staining intensity was positively correlated with GLI1 expression, an important component of SHH pathway, in NB tumor samples (S4 Table), which suggests HIF-1α as well as SHH signaling may play an important role in NB initiation or progression.

Indeed, we demonstrated that HIF-1α enhanced the proliferation and migration of NB cells under hypoxic rather than normoxic conditions. These abilities were reduced when HIF-1α was knocked down by Lenti-HIF-1α siRNA (Figs. 3–5). Noteworthy, a number of studies showed similar findings with ours. Two previous studies showed that specific blockade of HIF-lα inhibited the proliferation of lung cancer cells and pancreatic cancer cells under hypoxia rather than normoxia [29,30]. In another two studies, the authors indicated that HIF-1α knockdown can markedly reduce the migration ability of esophageal cancer cells and osteosarcoma cells under hypoxia [31,32]. It is known that, cells use oxygen-dependent metabolic pathway such as the tricarboxylic acid (TCA) cycle to produce energy under normoxia. However, they start using glycolysis as the primary mechanism of ATP production when oxygen is depleted. Many genes involved in glucose uptake and glycolysis have been identified as HIF-1 target genes, and could promote the growth and maintenance of cancer cells under hypoxic conditions [33–35]. This might be the reason why HIF-1α enhanced the proliferation and migration of NB cells under hypoxia rather than under normoxia. Interestingly, we also found that HIF-1α enhanced the invasive abilities of NB cells under both normoxia and hypoxia (Fig. 5). This finding is consistent with a recent study which evaluated the effect of HIF-1α on the invasion of lung cancer cells [29]. The possible explanation for this phenomenon is that HIF-1α enhances the expression of matrix metalloproteinases (MMPs) which can degrade most of the components of the extracellular matrix (ECM) and facilitate the invasion and metastasis of cancer cells [36,37].

In addition, to supplement our in vitro findings with in vivo data, we made xenografts of the HIF-1α overexpression or knockdown NB cells in nude mice. The analysis of the xenografts demonstrated a notable correlation between HIF-1α expression levels and tumor sizes, confirming that HIF-1α could promote xenograft growth (Fig. 9A and B). We also demonstrated that HIF-1α expression in xenografts was associated with increased vascularization (Fig. 9C), which might be involved in the HIF-1α-promoting NB tumor progression.

Whether the SHH pathway promotes NB development remains controversial. SHH signals were found to be critical for the growth and invasion of NB cells [20–22,38]. In our study, we showed that positive staining for SHH and GLI1 was associated with advanced stage, lymph node metastasis and poor differentiation in NB specimens via IHC analysis (Table 1). We further investigate whether the SHH pathway is involved in the HIF-1α-mediated positive regulation of cell growth, migration and invasion in NB cells under hypoxic conditions. GLI1 was knocked down by siRNA to block the SHH pathway in NB cells. Assays revealed that knockdown of GLI1 in NB cells led to decreased growth, migration and invasion abilities under hypoxia (Fig. 7). In model of xenograft, the HIF-1α-overexpressing xenograft showed strong staining for SHH pathway components, and the HIF-1α-silencing xenograft showed weak staining (Fig. 9C). These results suggested that HIF-1α resulted in de novo SHH synthesis, and thereafter triggering the SHH pathway. All of these data indicate that cell proliferation, migration and invasion of NB cells in response to HIF-1α are regulated, at least in part, through the SHH pathway.

Taken together, our findings support the SHH pathway as a regulator of HIF-1α-induced NB growth and progression and, possibly, as a novel target for NB therapeutics.

## Supporting Information

S1 Table Sequences of siRNAs targeting GLI1. (DOCX)

S2 Table Primers used for quantitative real-time PCR. (DOCX)

S3 Table Sequences of siRNAs targeting HIF-1α. (DOCX)

S4 Table HIF-1α and SHH signals expression and clinicopathologic characteristics of NBs. (DOCX)
