# Supplementary material for: HIF-1α Contributes to Proliferation and Invasiveness of Neuroblastoma Cells via SHH Signaling
Source: PLoS One. 2015 Mar 26;10(3):e0121115. doi: 10.1371/journal.pone.0121115 (PMC4374675; doi:10.1371/journal.pone.0121115)
Supplement: S1 Table — (DOCX) [file pone.0121115.s001.docx]

**S1 Table. Sequences of siRNAs targeting GLI1.**

| Name | Sequence |
| --- | --- |
| GLI1 siRNA1 | 5'- GCCCAGAUGAAUCACCAAATT-3' |
| GLI1 siRNA2 | 5'- GGCUCAGCUUGUGUGUAAUTT-3' |
| GLI1 siRNA3 | 5'- GUCCUCGACUUGAACAUUATT-3' |
| NC siRNA | 5'- UUCUCCGAACGUGUCACGUdTdT-3' |
