# Supplementary material for: HIF-1α Contributes to Proliferation and Invasiveness of Neuroblastoma Cells via SHH Signaling
Source: PLoS One. 2015 Mar 26;10(3):e0121115. doi: 10.1371/journal.pone.0121115 (PMC4374675; doi:10.1371/journal.pone.0121115)
Supplement: S2 Table — (DOCX) [file pone.0121115.s002.docx]

**S2 Table. Primers used for quantitative real-time PCR.**

| Pimer name | Sequence |
| --- | --- |
| SHH | Forward 5'- TCTGCTGCTAGTCCTCGTCT-3'  Reverse 5'- TGTCGGGGTTGTAATTGGGG-3' |
| PTCH1 | Forward 5'-CAAACTCCTGGTGCAAACCG-3' |
|  | Reverse 5'-CCGGGATTCTCAGCCTTGTT-3' |
| GLI1 | Forward 5'-CCTTGAGAACCTCAGGCTGG-3' |
|  | Reverse 5'-CCCCTGCATTGGGGTTGTAT-3' |
| β-actin | Forward 5'-CCTGTACGCCAACACAGTGC-3'  Reverse 5'-ATACTCCTGCTTGCTGATCC-3' |
