# Supplementary material for: HIF-1α Contributes to Proliferation and Invasiveness of Neuroblastoma Cells via SHH Signaling
Source: PLoS One. 2015 Mar 26;10(3):e0121115. doi: 10.1371/journal.pone.0121115 (PMC4374675; doi:10.1371/journal.pone.0121115)
Supplement: S3 Table — (DOCX) [file pone.0121115.s003.docx]

**S3 Table. Sequences of siRNAs targeting HIF-1α.**

| Name | Sequence |
| --- | --- |
| HIF-1α siRNA1 | 5'-GCTGACCAGTTATGATTGT-3' |
| HIF-1α siRNA2 | 5'-GCAGACTCAAATACAAGAA-3' |
| HIF-1α siRNA3 | 5'-TGTGAGTTCGCATCTTGAT-3' |
| NC siRNA | 5'-TTCTCCGAACGTGTCACG-3' |
