# Supplementary material for: HIF-1α Contributes to Proliferation and Invasiveness of Neuroblastoma Cells via SHH Signaling
Source: PLoS One. 2015 Mar 26;10(3):e0121115. doi: 10.1371/journal.pone.0121115 (PMC4374675; doi:10.1371/journal.pone.0121115)
Supplement: S4 Table — (DOCX) [file pone.0121115.s004.docx]

**S4 Table. HIF-1α and SHH signals expression and clinicopathologic characteristics of NBs.**

| Samples  No | Gender | Age | INSS stage | L[ymph node](http://dict.youdao.com/search?q=lymphatic&keyfrom=E2Ctranslation) [metastasis](http://dict.youdao.com/w/metastasis/) | Differentiation | HIF-1α | SHH | PTCH1 | GLI1 |
| --- | --- | --- | --- | --- | --- | --- | --- | --- | --- |
| 1 | M | 11M | I | No | Good | 0 | 0 | 0 | 0 |
| 2 | F | 2Y | I | No | Poor | 0 | 0 | 1 | 0 |
| 3 | M | 4M | I | No | Poor | 0 | 0 | 1 | 1 |
| 4 | F | 2Y | I | No | Good | 2 | 1 | 0 | 0 |
| 5 | F | 4M | I | No | Poor | 0 | 0 | 0 | 1 |
| 6 | M | 4Y | I | No | Good | 0 | 2 | 2 | 0 |
| 7 | F | 11Y | I | No | Good | 1 | 0 | 1 | 0 |
| 8 | M | 13M | I | No | Good | 1 | 0 | 1 | 1 |
| 9 | F | 15M | I | No | Poor | 1 | 2 | 0 | 0 |
| 10 | F | 2Y | I | No | Poor | 1 | 1 | 1 | 0 |
| 11 | M | 8M | I | No | Good | 0 | 1 | 0 | 0 |
| 12 | M | 15M | I | No | Poor | 0 | 0 | 0 | 1 |
| 13 | F | 6Y | I | No | Good | 0 | 0 | 0 | 0 |
| 14 | F | 3Y | I | No | Good | 0 | 0 | 0 | 0 |
| 15 | M | 18M | I | No | Good | 0 | 0 | 0 | 0 |
| 16 | M | 10M | I | No | Poor | 0 | 2 | 0 | 0 |
| 17 | M | 6Y | I | No | Good | 0 | 0 | 0 | 0 |
| 18 | M | 4Y | I | No | Good | 0 | 1 | 0 | 0 |
| 19 | F | 14M | II | No | Good | 0 | 0 | 1 | 0 |
| 20 | F | 2Y | II | Yes | Poor | 0 | 2 | 0 | 1 |
| 21 | F | 12M | II | No | Poor | 0 | 0 | 0 | 2 |
| 22 | F | 2Y | II | No | Good | 0 | 1 | 1 | 1 |
| 23 | F | 13M | II | No | Poor | 0 | 1 | 1 | 0 |
| 24 | M | 4Y | II | No | Good | 1 | 3 | 0 | 0 |
| 25 | F | 9M | II | No | Poor | 2 | 2 | 1 | 1 |
| 26 | F | 2Y | II | No | Good | 2 | 2 | 1 | 2 |
| 27 | F | 4Y | II | Yes | Poor | 0 | 0 | 1 | 1 |
| 28 | M | 18M | II | Yes | Good | 0 | 0 | 0 | 0 |
| 29 | M | 3Y | II | Yes | Poor | 0 | 0 | 0 | 1 |
| 30 | F | 9Y | II | No | Good | 0 | 0 | 1 | 1 |
| 31 | F | 18M | II | No | Good | 0 | 1 | 0 | 1 |
| 32 | M | 4Y | II | No | Poor | 1 | 1 | 2 | 2 |
| 33 | M | 8M | II | Yes | Poor | 1 | 1 | 1 | 1 |
| 34 | M | 2Y | II | No | Good | 1 | 0 | 0 | 1 |
| 35 | F | 8Y | II | No | Good | 1 | 0 | 1 | 1 |
| 36 | F | 2Y | III | Yes | Poor | 1 | 2 | 2 | 1 |
| 37 | F | 5Y | III | No | Poor | 1 | 2 | 0 | 1 |
| 38 | M | 3Y | III | Yes | Good | 1 | 1 | 1 | 1 |
| 39 | F | 4Y | III | No | Poor | 2 | 0 | 1 | 2 |
| 40 | F | 17M | III | No | Poor | 2 | 1 | 0 | 0 |
| 41 | F | 12M | III | No | Poor | 1 | 1 | 0 | 0 |
| 42 | F | 3Y | III | Yes | Poor | 1 | 2 | 0 | 2 |
| 43 | M | 8Y | III | Yes | Poor | 2 | 2 | 0 | 1 |
| 44 | M | 3Y | III | No | Poor | 3 | 1 | 0 | 0 |
| 45 | F | 6Y | III | No | Good | 1 | 2 | 0 | 0 |
| 46 | F | 2Y | III | Yes | Good | 0 | 1 | 0 | 1 |
| 47 | F | 2Y | III | No | Poor | 2 | 0 | 0 | 2 |
| 48 | M | 3Y | IV | Yes | Poor | 3 | 3 | 1 | 3 |
| 49 | M | 3Y | IV | Yes | Poor | 2 | 2 | 2 | 1 |
| 50 | M | 2Y | IV | Yes | Good | 0 | 1 | 0 | 0 |
| 51 | M | 3Y | IV | Yes | Poor | 1 | 0 | 1 | 0 |
| 52 | M | 4M | IV | Yes | Poor | 1 | 0 | 1 | 0 |
| 53 | M | 3Y | IV | Yes | Poor | 1 | 2 | 0 | 3 |
| 54 | F | 6Y | IV | Yes | Poor | 1 | 2 | 2 | 1 |
| 55 | M | 13Y | IV | Yes | Good | 2 | 3 | 1 | 0 |
| 56 | M | 3Y | IV | Yes | Poor | 1 | 2 | 1 | 0 |
| 57 | M | 4Y | IV | Yes | Poor | 0 | 0 | 1 | 0 |
| 58 | M | 19M | IV | Yes | Good | 3 | 2 | 0 | 2 |
| 59 | M | 4Y | IV | Yes | Good | 0 | 0 | 1 | 1 |
| 60 | M | 4Y | IV | Yes | Poor | 1 | 2 | 0 | 2 |
| 61 | M | 3Y | IV | Yes | Poor | 2 | 3 | 2 | 3 |
| 62 | F | 3Y | IV | Yes | Poor | 1 | 1 | 1 | 1 |
| 63 | F | 3Y | IV | Yes | Good | 0 | 2 | 0 | 0 |
| 64 | M | 9Y | IV | Yes | Poor | 1 | 2 | 0 | 0 |
| 65 | F | 2Y | IV | Yes | Good | 0 | 3 | 0 | 1 |
| 66 | M | 14M | IV | Yes | Poor | 2 | 3 | 1 | 3 |
| 67 | M | 3Y | IV | Yes | Poor | 2 | 3 | 0 | 3 |
| 68 | F | 4Y | IV | Yes | Good | 1 | 1 | 1 | 0 |
| 69 | M | 3Y | IV | Yes | Good | 0 | 2 | 0 | 0 |
| 70 | M | 3Y | IV | Yes | Poor | 1 | 3 | 2 | 1 |
| 71 | M | 15M | IV | Yes | Poor | 1 | 1 | 0 | 2 |
